# Craniodental and Postcranial Characters of Non-Avian Dinosauria Often Imply Different Trees

**DOI:** 10.1093/sysbio/syz077

**Published:** 2019-11-26

**Authors:** Yimeng Li, Marcello Ruta, Matthew A Wills

**Affiliations:** 1 Department of Biology & Biochemistry, The Milner Centre for Evolution, The University of Bath, The Avenue, Claverton Down, Bath BA2 7AY, UK; 2 School of Life Sciences, University of Lincoln, Joseph Banks Laboratories, Green Lane, Lincoln LN6 7DL, UK

## Abstract

Despite the increasing importance of molecular sequence data, morphology still makes an important contribution to resolving the phylogeny of many groups, and is the only source of data for most fossils. Most systematists sample morphological characters as broadly as possible on the principle of total evidence. However, it is not uncommon for sampling to be focused on particular aspects of anatomy, either because characters therein are believed to be more informative, or because preservation biases restrict what is available. Empirically, the optimal trees from partitions of morphological data sets often represent significantly different hypotheses of relationships. Previous work on hard-part versus soft-part characters across animal phyla revealed significant differences in about a half of sampled studies. Similarly, studies of the craniodental versus postcranial characters of vertebrates revealed significantly different trees in about one-third of cases, with the highest rates observed in non-avian dinosaurs. We test whether this is a generality here with a much larger sample of 81 published data matrices across all major dinosaur groups. Using the incongruence length difference test and two variants of the incongruence relationship difference test, we found significant incongruence in about 50% of cases. Incongruence is not uniformly distributed across major dinosaur clades, being highest (63%) in Theropoda and lowest (25%) in Thyreophora. As in previous studies, our partition tests show some sensitivity to matrix dimensions and the amount and distribution of missing entries. Levels of homoplasy and retained synapomorphy are similar between partitions, such that incongruence must partly reflect differences in *patterns* of homoplasy between partitions, which may itself be a function of modularity and mosaic evolution. Finally, we implement new tests to determine which partition yields trees most similar to those from the entire matrix. Despite no bias across dinosaurs overall, there are striking differences between major groups. The craniodental characters of Ornithischia and the postcranial characters of Saurischia yield trees most similar to the “total evidence” trees derived from the entire matrix. Trees from these same character partitions also tend to be most stratigraphically congruent: a mutual consilience suggesting that those partitions yield more accurate trees. [Dinosauria; homoplasy; partition homogeneity.]

The fossil record is notoriously incomplete, not only in terms of diversity and species richness (Verriere et al. 2016; Davies et al. 2017; Tutin and Butler 2017), but also with respect to stratigraphy (Maxwell and Benton 1990; Dunhill et al. 2012; Brocklehurst and Froebisch 2014; O’Connor and Wills 2016; Verriere et al. 2016) paleobiogeography (Lieberman 2002; Ksepka and Boyd 2012; Davies et al. 2017), paleoecology (Stanley et al. 1989), and behavior (Jablonski 2005; Hsiang et al. 2015; Daley and Drage 2016; Fan et al. 2017). However, it is organismal incompleteness—the selective preservation of tissues and body regions—that impinges most directly on attempts to infer phylogeny (Kearney and Clark 2003; Cobbett et al. 2007; Sansom 2015). The fossil record of non-avian dinosaurs mostly comprises bones and other hard parts (Wills et al. 2008; Mannion and Upchurch 2010), but there are further biases towards the preservation of more heavily mineralized and massive elements (e.g., limb bones) at the expense of more frangible and delicate structures (e.g., skulls). Inferred relationships may differ substantially depending upon which subsets of characters are used, but paleontologists may nonetheless wish to infer the relationships of dinosaurs described from partial skeletal material. Nevertheless, previous studies have demonstrated that trees of dinosaurs have strikingly better stratigraphic congruence than most other groups of vertebrates (Wills et al. 2008), and certainly better than most invertebrate groups (O’Connor and Wills 2016). Where stratigraphic congruence is high overall, it offers an ancillary criterion for choosing between equally optimal or otherwise competing sets of trees, as well as the phylogenetic informativeness of the data underpinning them (Huelsenbeck 1994; Wills 1998; Wills et al. 2009; O’Connor and Zhou 2013; O’Connor and Wills 2016). Non-avian dinosaurs also have the advantage—for this test at least—that they are all extinct and therefore (by definition) all have a fossil record (Benton 2008).

We therefore address five related questions using a sample of 81 cladistic taxon-character matrices published between 2011 and 2017 (Lloyd 2017) (Supplementary Materials S1–S3 available on Dryad at http://dx.doi.org/10.5061/dryad.gxd2547gj), each comprising both craniodental and postcranial characters. This represents a sample of the recent phylogenetic literature across major dinosaur groups, and minimizes the overlap of taxa and characters between matrices (see below).

Firstly, do levels of homoplasy differ between characters of the skull and dentition on the one hand, and characters pertaining to the body on the other? Any such difference might be used to argue for the “superiority” of one body region over the other for phylogenetic inference (Pettigrew 1991; Sanchez-Villagra and Williams 1998; Williams 2007; Song and Bucheli 2010; Mounce et al. 2016; Parker 2016). Secondly, are the most parsimonious trees (MPTs) inferred from craniodental and postcranial character partitions significantly different (Mounce et al. 2016; Sansom et al. 2017)? We address this using established (incongruence length difference [ILD]; Farris et al. 1994) and more recent (Mounce et al. 2016; Sansom et al. 2017) tests. Thirdly, are there differences in the incidence of significant craniodental/postcranial incongruence across major taxonomic groups of non-avian Dinosauria? Fourthly, are the tree(s) inferred from craniodental characters or the tree(s) derived from postcranial characters more similar to those derived from the entire matrix (with the latter being used as a proxy for the “true” phylogeny)? We address this using a novel test that resamples from the partitions and the entire matrix in order to control for differences in the number of characters in each partition. Fifthly, does the stratigraphic consistency of trees inferred from craniodental and postcranial character data differ, and are the trees with greatest stratigraphic consistency also the most similar to total evidence trees?

Suites of morphological characters are often functionally and developmentally integrated into modules (Clarke and Middleton 2008; Klingenberg 2008; Lü et al. 2010) that can be subject to different selection pressures and consequently evolve at different speeds (Lü et al. 2010; Parker 2016). This has consequences for the rate at which new character states are utilized and the subsequent exhaustion of character space (Wagner 1995, 1997; Oyston et al. 2015, 2016), resulting in different levels of homoplasy. For example, it has been shown that the dental characters of mammals are particularly labile and prone to convergence/reversal (Sanchez-Villagra and Williams 1998; Sansom et al. 2017), which is explicable in terms of the strong functional and biomechanical constraints upon the form and arrangement of teeth. This particular bias is unfortunate given the predominance of teeth in the mammal fossil record. More generally, the craniodental and postcranial characters of vertebrates have been shown to contain significantly incongruent signals about one time in three (Mounce et al. 2016). Moreover, it is possible that incongruence is partly a function of the extent to which the skull and the rest of the body are biomechanically decoupled (Ji et al. 1999). Fishes (lacking a functional neck) typically show integration, while the most striking incongruence has been observed in some of the long-necked dinosaur groups.

As a prerequisite for combining data in early, multigene molecular analyses, systematists commonly checked for homogeneity of signal across loci using a variety of partition tests (Templeton 1983; Rodrigo et al. 1993; Farris et al. 1994). This practice is rarely implemented nowadays, and for principally three reasons (Cunningham 1997). Firstly, as analyses of increasingly large numbers of genes graded into phylogenomic studies, the concept of the contingent inclusion of individual genes became largely obsolete. Secondly, more advanced analytical methods were developed that allow heterogeneous rates across sites and branches to be modeled rigorously (Damgaard 2012). Thirdly, a consensus emerged amongst systematists in favor of the simultaneous analysis of all available character data, on the principle of “total evidence” (Kluge 1989), not least because of “hidden support”. This last is the phenomenon whereby signals that are weak within particular partitions of the data may be common to many (or all) such partitions, such that they become the dominant signal when all partitions are analyzed together (Kluge 1989; Gatesy et al. 1999; Gatesy and Arctander 2000; Wahlberg et al. 2005; O’Leary and Gatesy 2008; Padial et al. 2010; Damgaard 2012; Mounce et al. 2016). Whereas progressively larger sequence matrices originally accreted through research time in a combinatorial manner (begging the question of heterogeneity), morphological matrices have almost invariably been generated and analyzed holistically, such that the question of partition heterogeneity has rarely arisen. The recent emphasis on developing more plausible models of morphological character state evolution (Wright et al. 2016) has spurred the development of approaches capable of automatically identifying partitions within morphological data sets (Lanfear et al. 2017). There is also evidence that partitioning morphological character data can better constrain error bars in morphological clock analyses (Caldas and Schrago 2019).

Despite the ascendance of molecular phylogenetics, morphological character data can still contribute to our understanding of the relationships of many groups (Houde 1994; Wiens 2004; O’Leary and Gatesy 2008; Nicolalde-Morejon et al. 2009; Gainett et al. 2014; Lopardo and Hormiga 2015). Moreover, for extinct and particularly for fossil groups, morphology is usually the only direct source of phylogenetic data, notwithstanding exceptional cases utilizing fossil DNA (Dabney et al. 2013; Shapiro and Hofreiter 2014; Orlando et al. 2015).

## Materials and Methods

### Data Sets

**Figure 1.**
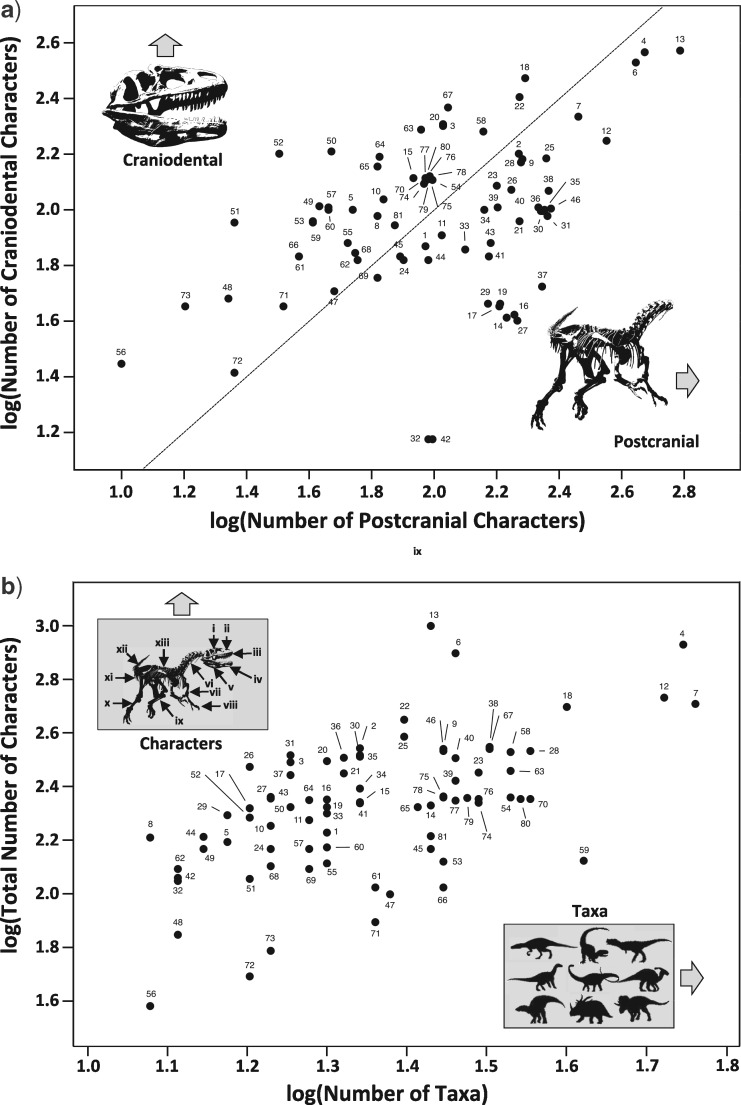
Scatter plots of data matrix and data matrix partition dimensions across our 81 analyzed matrices of non-avian Dinosauria. **a**) log(number of craniodental characters) against log(number of postcranial characters). The dotted line indicates the 1:1 slope. Points above this line have a higher proportion of craniodental characters, while points below have a higher proportion of postcranial characters. Numbers of craniodental and postcranial characters are not significantly different according to a paired Wilcoxon test ($P=$ 0.1343). **b**) log(total number of characters) against log(total number of taxa). Source papers are as follows: *Theropoda* 1. Allain et al. (2012), 2. Araújo et al. (2013), 3. Brusatte and Benson (2013), 4. Brusatte et al. (2014), 5. Canale et al. (2015), 6. Cau et al. (2012), 7. Choiniere et al. (2014), 8. Eddy and Clarke (2011), 9. Evers et al. (2015), 10. Fanti et al. (2012), 11. Farke and Sertich (2013), 12. Foth et al. (2014), 13. Godefroit et al. (2013), 14. Hu et al. (2015), 15. Lamanna et al. (2014), 16. Lee et al. (2014), 17. Li et al. (2014), 18. Loewen et al. (2013), 19. Longrich et al. (2011), 20. Lu et al. (2014), 21. Novas et al. (2013), 22. Parsons and Parsons (2015), 23. Porfiri et al. (2014), 24. Sanchez-Hernandez and Benton (2014), 25. Senter et al. (2012), 26. Tortosa et al. (2014), 27. Wang et al. (2015), 28. Zanno and Makovicky (2013), 29. Zhou et al. (2014), *Sauropodomorpha* 30. Carballido and Sander (2014), 31. Carballido et al. (2015), 32. D’Emic (2013), 33. Fanti et al. (2015), 34. Gorscak et al. (2014), 35. Lacovara et al. (2014), 36. Li et al. (2014), 37. Mannion et al. (2013), 38. McPhee et al. (2015), 39. Pol et al. (2011a), 40. Rauhut et al. (2015), 41. Rubilar-Rogers et al. (2012), 42. Saegusa and Ikeda (2014), 43. Santucci and De Arruda-Campos (2011), 44. Tschopp and Mateus (2013), 45. Wilson and Allain (2015), 46. Xing et al. (2015), *Cerapoda* 47. Evans and Ryan (2015), 48. Farke et al. (2011), 49. Farke et al. (2014), 50. Han et al. (2015), 51. Longrich (2011), 52. Longrich (2014), *Ornithopoda* 53. Boyd and Pagnac (2015), 54. Boyd (2015), 55. Brown et al. (2011), 56. Evans et al. (2013), 57. Godefroit et al. (2012), 58. He et al. (2015), 59. McDonald (2012), 60. McGarrity et al. (2013), 61. Norman (2015), 62. Norman et al. (2011), 63. Prieto-Marquez and Wagner (2013), 64. Prieto-Marquez (2014), 65. Prieto-Marquez et al. (2013), 66. Shibata et al. (2015), 67. Xing et al. (2014), *Thyreophora* 68. Arbour and Currie (2013), 69. Arbour et al. (2014), 70. Barrett et al. (2014), 71. Burns and Currie (2014a), 72. Burns and Currie (2014b), 73. Burns et al. (2011), 74. Butler et al. (2011), 75. Coria et al. (2013), 76. Godefroit et al. (2014), 77. Han et al. (2012), 78. Osi et al. (2012), 79. Pol et al. (2011b), 80. Ruiz-Omenaca et al. (2012), 81. Thompson et al. (2012). *Allosaurus* images in panels a and b modified from: https://commons.wikimedia.org/wiki/File:Allosaurus_AMNH_lobby_(white_background).jpg. Other dinosaur silhouettes drawn by Yimeng Li.

The character matrices utilized here were obtained from peer-reviewed papers published between 2011 and 2017. We utilized Graeme Lloyd’s online list of published matrices (Wright et al. 2016; Lloyd 2017) in order to sample all major dinosaur groups, including matrices of varying dimensions. Character lists and descriptions were then obtained from the original publications. We initially included 104 matrices, but these were further checked for overlap since systematists often repurpose data or otherwise add modest numbers of novel taxa and sometimes characters to existing studies. In order to remove any such pseudoreplication from our sample, each data set was compared with every other, and for each pair the number of matrix cells in common (replicated taxa and characters) was expressed as a percentage of the total number of cells in the smaller of the two matrices. For pairs with 20% or more overlap, the least inclusive (or otherwise the oldest) was removed from consideration, reducing our sample to 81 data sets (see Supplementary Table 1 available on Dryad for the percentage of character and species overlap between all pairs). We note that a comparable approach was used in the data compiled by Lloyd (Wright et al. 2016). Character lists were then used to define partitions. The “craniodental” partition included all characters pertaining to the skull and dentition. The “postcranial” partition encompassed all characters of the vertebral column, girdles, and limbs. Small numbers of characters pertaining to features that could not be partitioned in this way (e.g., those pertaining to the integument, feathers, eggs, or ecology) were removed from consideration. Poorly known taxa, or those that are otherwise scored for only a small number of characters, can be highly unstable within parsimony trees. This, in turn, can result in large numbers of MPTs, prohibitively extending search times, and yielding poorly resolved consensus trees (Wilkinson 1995; Mounce et al. 2016). Where such complications were found in our analyses, the matrix was edited by removing taxa with more than 40% of characters scored as missing (“?”) or nonapplicable (“-”) in either partition. Any characters rendered uninformative or invariant by this process were also deleted (Wiens 1998). On average, 20 taxa and 18 characters were removed from each data set in this way, equating to 33% of taxa and 7% of characters. For a list of characters and taxa discounted, see Supplementary Material S2 available on Dryad. We acknowledge that these procedures modify matrices from their original published form, such that no inferences should be made concerning the quality of the original data sets. Moreover, the original matrices were assembled for holistic (rather than partitioned) analysis, and we deviate from the purposes of the original authors in this respect. Matrices were manipulated using Mesquite Version 3.04 (build 725) (Maddison and Maddison 2015) for Macintosh. The resulting sample of 81 matrices contained an average of 26 taxa scored for a mean of 115 craniodental and 133 postcranial characters (distributions of numbers of characters and taxa are illustrated in Fig. 1).

We also acknowledge that Bayesian methods are increasingly being applied to morphological character data (Pollitt et al. 2005; O’Reilly et al. 2016), but maximum parsimony is still the most widely implemented approach. From our sample of 81 matrices of non-avian dinosaurs, all were analyzed by the original authors using parsimony, while 6 were also analyzed using Bayesian methods (see sources marked with “*” in Table 1).

**Table 1.** Summary of all partition tests and statistics for all 81 partitioned data sets

References	Ref. no.	Group	Number of craniodental characters	Number of postcranial characters	Taxa	Taxa removed	test $P$ value	${\rm ILD}_{{{{\rm NND}+{\rm RF}}}}$ $P$ value	${\rm IRD}_{{{{\rm MR}+{\rm RF}}}}$ $P$ value	${\rm IRD}_{{{{\rm NND}+{\rm matching}}}}$ $P$ value	${\rm IRD}_{{{{\rm MR}+{\rm matching}}}}$ $P$ value	Craniodental missing %	Postcranial missing %	Total missing %	Craniodental CI	Craniodental RI	Postcranial CI	Postcranial RI	Craniodental or postcranial most similar to entire	Craniodental or postcranial $P$ value
Allain et al. (2012)	1	Theropoda	74	94	20	9	0.140	0.11	0.04	0.07	0.04	16.22	19.47	18.04	0.517	0.667	0.585	0.762	Post	0.00000
Araújo et al. (2013)	2	Theropoda	159	187	22	42	0.011	0.01	0.01	0.03	0.03	30.16	26.15	27.99	0.506	0.603	0.590	0.729	Post	0.03120
Brusatte and Benson (2013)	3	Theropoda	200	107	18	16	0.164	0.01	0.01	0.02	0.05	23.67	34.53	27.46	0.681	0.835	0.665	0.846	Cranio	0.00000
Brusatte et al. (2014)	4	Theropoda	368	473	56	95	0.054	0.01	0.01	0.01	0.01	49.53	39.33	43.79	0.458	0.762	0.422	0.578	Post	0.16930
Canale et al. (2015)	5	Theropoda	100	55	15	1	0.610	0.01	0.21	0.05	0.07	35.80	39.27	37.03	0.667	0.762	0.624	0.658	Cranio	0.00000
Cau et al. (2012)	6	Theropoda	338	443	29	8	0.031	0.03	0.03	0.02	0.01	38.32	45.39	42.33	0.393	0.494	0.433	0.519	Post	0.00000
Choiniere et al. (2014)	7	Theropoda	216	290	58	56	0.001	0.01	0.01	0.01	0.01	48.83	46.83	47.68	0.289	0.582	0.303	0.601	Post	0.00012
Eddy and Clarke (2011)	8	Theropoda	95	66	12	10	0.032	0.18	0.23	0.11	0.11	26.93	35.35	30.38	0.629	0.670	0.705	0.730	Post	0.00242
Evers et al. (2015)	9	Theropoda	152	192	28	36	0.106	0.01	0.01	0.01	0.01	46.20	43.42	44.65	0.434	0.672	0.464	0.716	Cranio	0.52290
Fanti et al. (2012)	10	Theropoda	109	69	17	2	0.001	0.01	0.02	0.03	0.03	31.41	37.00	33.58	0.750	0.844	0.555	0.599	Cranio	0.00000
Farke and Sertich (2013)	11	Theropoda	81	106	19	11	0.998	0.02	0.01	0.01	0.01	46.52	43.64	44.89	0.733	0.856	0.587	0.677	Cranio	0.01551
Foth et al. (2014)	12	Theropoda	177	357	53	79	0.001	0.01	0.01	0.01	0.08	39.20	35.90	36.99	0.411	0.768	0.392	0.741	Post	0.24930
Godefroit et al. (2013)	13	Theropoda	373	614	27	74	0.001	0.01	0.01	0.01	0.01	41.22	47.80	45.31	0.465	0.522	0.521	0.578	Cranio	0.89400
Hu et al. (2015)	14	Theropoda	41	171	27	35	0.231	0.63	0.76	0.02	0.02	57.18	33.10	37.76	0.676	0.783	0.482	0.634	Post	0.05020
Lamanna et al. (2014)	15	Theropoda	130	86	22	19	0.001	0.04	0.01	0.03	0.02	34.44	45.40	38.80	0.644	0.771	0.531	0.591	Cranio	0.00000
Lee et al. (2014) $^{*}$	16	Theropoda	42	181	20	45	0.165	0.35	0.68	0.38	0.75	37.50	33.50	34.25	0.690	0.821	0.614	0.732	Cranio	0.00000
Li et al. (2014a)	17	Theropoda	45	162	16	20	0.372	0.84	0.85	0.67	0.70	30.42	16.51	19.53	0.545	0.609	0.464	0.534	Cranio	0.00398
Loewen et al. (2013)	18	Theropoda	297	196	40	0	0.122	0.08	0.06	0.05	0.12	29.69	29.96	29.80	0.409	0.760	0.424	0.771	Post	0.00000
Longrich (2011)	19	Theropoda	46	163	20	26	0.002	0.01	0.01	0.01	0.03	42.50	21.53	26.15	0.815	0.908	0.635	0.791	Post	0.02580
Lu et al. (2014)	20	Theropoda	203	107	20	5	0.233	0.01	0.01	0.01	0.01	25.15	38.78	29.85	0.663	0.835	0.654	0.844	Cranio	0.00000
Novas et al. (2013)	21	Theropoda	91	188	21	24	0.475	0.03	0.05	0.01	0.09	21.40	30.37	27.44	0.482	0.653	0.514	0.666	Post	0.03884
Parsons and Parsons (2015)	22	Theropoda	254	188	25	93	0.016	0.01	0.01	0.03	0.02	24.41	23.74	24.13	0.552	0.782	0.635	0.833	Cranio	0.03490
Porfiri et al. (2014)	23	Theropoda	122	159	31	14	0.374	0.04	0.04	0.38	0.31	35.25	37.49	36.52	0.399	0.633	0.437	0.670	Post	0.14770
Sanchez-Hernandez and Benton (2014)	24	Theropoda	68	78	17	6	1.000	0.01	0.05	0.01	0.01	45.16	44.34	44.72	0.755	0.866	0.630	0.688	Cranio	0.00000
Senter et al. (2012)	25	Theropoda	153	229	25	85	0.021	0.04	0.06	0.01	0.01	20.73	17.83	18.99	0.658	0.860	0.620	0.818	Post	0.00000
Tortosa et al. (2014)	26	Theropoda	118	177	16	24	0.017	0.01	0.01	0.01	0.01	44.60	34.03	38.26	0.782	0.833	0.602	0.661	Post	0.00000
Wang et al. (2015)	27	Theropoda	40	185	17	41	0.018	0.18	0.51	0.17	0.13	43.38	30.84	33.07	0.712	0.784	0.655	0.710	Cranio	0.00038
Zanno and Makovicky (2013)	28	Theropoda	148	190	36	26	0.080	0.05	0.23	0.05	0.04	42.59	33.81	37.65	0.447	0.656	0.473	0.711	Post	0.00000
Zhou et al. (2014)	29	Theropoda	46	149	15	18	0.001	0.02	0.20	0.03	0.12	26.96	15.84	18.46	0.775	0.857	0.746	0.811	Post	0.05590
Carballido and Sander (2014)	30	Sauropod.	99	223	22	52	0.002	0.10	0.09	0.17	0.16	33.47	29.03	30.40	0.663	0.785	0.570	0.653	Cranio	0.00000
Carballido et al. (2015)	31	Sauropod.	95	231	18	55	0.041	0.05	0.08	0.04	0.05	29.07	20.06	22.69	0.647	0.720	0.586	0.598	Post	0.06600
D’Emic (2013)	32	Sauropod.	15	96	13	12	0.018	0.05	0.06	0.03	0.08	23.60	18.20	18.93	0.727	0.807	0.777	0.859	Post	0.00006
Fanti et al. (2015) $^{*}$	33	Sauropod.	72	126	20	0	0.466	0.01	0.01	0.16	0.11	41.32	27.18	32.32	0.711	0.819	0.515	0.633	Post	0.00000
Gorscak et al. (2014) $^{*}$	34	Sauropod.	100	145	22	13	0.074	0.02	0.02	0.02	0.01	40.09	31.57	35.05	0.669	0.761	0.668	0.756	Post	0.00000
Lacovara et al. (2014)	35	Sauropod.	100	226	22	53	0.009	0.03	0.03	0.01	0.02	34.40	27.82	29.84	0.667	0.777	0.574	0.668	Post	0.29510
Li et al. (2014b)	36	Sauropod.	99	220	21	50	0.020	0.34	0.24	0.26	0.28	32.13	26.02	27.92	0.669	0.778	0.575	0.656	Cranio	0.00000
Mannion et al. (2013)	37	Sauropod.	53	222	18	45	0.129	0.06	0.20	0.03	0.03	39.41	22.87	26.06	0.576	0.669	0.402	0.511	Cranio	0.13200
McPhee et al. (2015)	38	Sauropod.	117	233	32	22	0.496	0.04	0.20	0.16	0.15	30.90	20.53	24.00	0.423	0.692	0.389	0.652	Post	0.00000
Pol et al. (2011a)	39	Sauropod.	102	160	29	21	0.120	0.52	0.58	0.37	0.29	37.59	36.12	36.69	0.625	0.793	0.667	0.798	Cranio	0.00714
Rauhut et al. (2015)	40	Sauropod.	102	216	29	43	0.677	0.01	0.01	0.01	0.01	47.63	26.48	33.26	0.603	0.748	0.476	0.705	Post	0.00000
Rubilar-Rogers et al. (2012)	41	Sauropod.	68	150	22	8	0.080	0.01	0.01	0.01	0.01	46.19	24.94	31.57	0.706	0.787	0.661	0.801	Post	0.00000
Saegusa and Ikeda (2014)	42	Sauropod.	15	99	13	16	0.025	0.06	0.04	0.01	0.03	23.60	18.41	19.09	0.727	0.807	0.780	0.859	Post	0.00135
Santucci and De Arruda-Campos (2011)	43	Sauropod.	76	152	17	21	0.010	0.01	0.01	0.01	0.03	32.20	21.36	24.97	0.739	0.778	0.705	0.769	Post	0.00000
Tschopp and Mateus (2013)	44	Sauropod.	66	96	14	0	0.131	0.12	0.29	0.04	0.32	47.29	30.73	37.48	0.696	0.663	0.582	0.618	Post	0.00000
Wilson and Allain (2015)	45	Sauropod.	66	80	27	0	0.263	0.01	0.01	0.01	0.01	31.47	15.38	22.65	0.868	0.911	0.664	0.768	Post	0.00000
Xing et al. (2015)	46	Sauropod.	101	237	28	17	0.096	0.01	0.01	0.01	0.01	22.60	32.69	29.67	0.557	0.714	0.481	0.663	Post	0.00000
Evans and Ryan (2015)	47	Cerapoda	51	48	24	1	0.078	0.04	0.03	0.01	0.01	29.17	43.66	36.20	0.647	0.827	0.764	0.844	Cranio	0.00000
Farke et al. (2011)	48	Cerapoda	48	22	13	5	0.796	0.10	0.71	0.04	0.47	16.50	31.10	21.09	0.739	0.786	0.852	0.852	Cranio	0.07588
Farke et al. (2014)	49	Cerapoda	103	43	14	19	0.082	0.04	0.22	0.03	0.01	20.94	20.59	20.84	0.750	0.841	0.746	0.837	Cranio	0.00000
Han et al. (2015)	50	Cerapoda	162	47	18	19	0.207	0.02	0.62	0.22	0.93	17.18	20.80	17.99	0.594	0.778	0.680	0.831	Cranio	0.00545
Longrich (2011)	51	Cerapoda	90	23	16	6	1.000	0.47	0.45	0.43	0.41	17.00	27.00	19.04	0.798	0.898	1.000	1.000	Post	0.00000
Longrich (2014)	52	Cerapoda	159	32	16	19	0.727	0.05	0.09	0.02	0.31	17.33	33.98	20.12	0.745	0.810	1.000	1.000	Cranio	0.00086
Boyd (2015) $^{*}$	53	Ornithopoda	90	41	28	40	0.028	0.07	0.01	0.02	0.01	25.00	27.44	25.76	0.571	0.789	0.548	0.799	Cranio	0.00000
Boyd (2015)	54	Ornithopoda	128	99	34	65	0.814	0.85	0.74	0.59	0.60	40.72	31.70	36.79	0.370	0.606	0.421	0.648	Post	0.00004
Brown et al. (2011)	55	Ornithopoda	76	53	20	3	0.181	0.39	0.46	0.21	0.38	27.43	23.11	25.66	0.519	0.688	0.503	0.654	Cranio	0.72770
Evans et al. (2013)	56	Ornithopoda	28	10	12	6	0.993	0.02	0.07	0.07	0.37	15.18	46.70	23.47	0.679	0.730	0.917	0.909	Cranio	0.20780
Godefroit et al. (2012)	57	Ornithopoda	100	46	19	2	0.108	0.02	0.01	0.02	0.02	18.84	30.32	22.46	0.688	0.777	0.678	0.767	Cranio	0.00000
He et al. (2015)	58	Ornithopoda	191	144	34	27	0.007	0.68	0.49	0.83	0.71	22.16	24.04	22.97	0.428	0.665	0.460	0.686	Post	0.14160
McDonald (2012)	59	Ornithopoda	91	41	42	25	0.285	0.04	0.03	0.13	0.15	21.89	24.00	22.55	0.596	0.798	0.596	0.811	Cranio	0.00000
McGarrity et al. (2013)	60	Ornithopoda	102	46	20	2	0.202	0.01	0.01	0.01	0.01	19.12	33.80	23.68	0.676	0.773	0.678	0.767	Cranio	0.00000
Norman (2015)	61	Ornithopoda	68	37	23	4	0.772	0.97	0.98	0.97	0.96	10.29	17.63	12.88	0.591	0.781	0.654	0.841	Cranio	0.64860
Norman et al. (2011)	62	Ornithopoda	66	57	13	1	0.090	0.12	0.23	0.11	0.15	12.82	18.89	15.63	0.566	0.559	0.631	0.648	Post	0.01156
Prieto-Marquez et al. (2013)	63	Ornithopoda	194	91	34	17	0.303	0.69	0.68	0.39	0.32	27.00	20.49	24.92	0.574	0.806	0.484	0.759	Cranio	0.02466
Prieto-Marquez (2014)	64	Ornithopoda	155	67	19	15	0.088	0.34	0.83	0.12	0.37	21.59	16.10	19.93	0.723	0.775	0.601	0.707	Post	0.22000
Prieto-Marquez and Wagner (2013) $^{*}$	65	Ornithopoda	143	66	26	8	0.475	0.01	0.03	0.14	0.43	27.65	29.37	28.19	0.597	0.780	0.558	0.685	Cranio	0.00014
Shibata et al. (2015)	66	Ornithopoda	68	37	28	0	0.760	0.95	1.00	0.96	1.00	10.29	17.62	12.87	0.591	0.781	0.654	0.841	Post	0.03930
Xing et al. (2014) $^{*}$	67	Ornithopoda	233	111	32	30	0.645	0.16	0.60	0.06	0.21	14.06	11.09	13.10	0.636	0.881	0.599	0.879	Cranio	0.00000
Arbour and Currie (2013)	68	Thyreophora	70	56	17	1	0.436	0.11	0.73	0.58	0.83	20.84	29.05	24.49	0.569	0.720	0.709	0.764	Cranio	0.04001
Arbour et al. (2014)	69	Thyreophora	57	66	19	1	0.460	0.14	0.31	0.11	0.08	22.90	44.82	34.66	0.546	0.721	0.673	0.757	Cranio	0.00000
Barrett et al. (2014)	70	Thyreophora	130	94	36	19	0.240	0.06	0.05	0.29	0.25	22.54	29.28	25.37	0.453	0.684	0.519	0.732	Post	0.02561
Burns and Currie (2014a)	71	Thyreophora	45	33	23	0	0.761	0.01	0.01	0.05	0.09	16.14	45.85	28.71	0.452	0.696	0.648	0.731	Cranio	0.00005
Burns and Currie (2014b)	72	Thyreophora	26	23	16	0	0.002	0.21	0.08	0.09	0.06	13.94	32.07	22.45	0.652	0.826	0.682	0.741	Cranio	0.00000
Burns et al. (2011)	73	Thyreophora	45	16	17	0	0.426	0.29	0.75	0.24	0.61	12.80	36.76	19.08	0.548	0.710	0.714	0.784	Cranio	0.05312
Butler et al. (2011)	74	Thyreophora	124	93	31	20	0.234	0.03	0.04	0.51	0.69	22.11	23.93	22.89	0.496	0.675	0.538	0.722	Post	0.00047
Coria et al. (2013)	75	Thyreophora	130	97	28	24	0.022	0.03	0.15	0.17	0.22	19.86	20.95	20.33	0.520	0.679	0.557	0.746	Post	0.03140
Godefroit et al. (2014)	76	Thyreophora	130	94	31	24	0.041	0.02	0.02	0.38	0.48	19.35	21.17	20.11	0.481	0.659	0.512	0.697	Post	0.01808
Han et al. (2012)	77	Thyreophora	127	94	29	25	0.137	0.07	0.04	0.24	0.12	18.06	18.56	18.27	0.511	0.688	0.532	0.713	Post	0.17900
Osi et al. (2012)	78	Thyreophora	132	97	28	30	0.313	0.03	0.03	0.01	0.02	18.56	18.00	18.32	0.435	0.671	0.509	0.707	Post	0.04740
Pol et al. (2011b)	79	Thyreophora	129	97	30	21	0.020	0.01	0.03	0.09	0.11	19.92	26.08	22.56	0.524	0.669	0.557	0.736	Post	0.01531
Ruiz-Omenaca et al. (2012)	80	Thyreophora	130	94	35	18	0.641	0.04	0.01	0.23	0.05	20.68	30.67	24.87	0.455	0.687	0.535	0.731	Cranio	0.00000
Thompson et al. (2012)	81	Thyreophora	88	75	27	24	0.294	0.09	0.13	0.02	0.11	24.45	35.80	29.67	0.445	0.729	0.454	0.675	Cranio	0.00000

IRD tests are based either upon the mean tree-to-tree distances between nearest neighbors (NND) or the distance between majority rule (plus compatible grouping) trees (MR). The tree-to-tree distance metric used is either the Robinson–Foulds (symmetrical difference) distance (RF) or the matching distance (matching). CI and RI refer to ensemble consistency and retention indices, respectively. “Craniodental or postcranial most similar to entire” indicates which mean nearest neighbor matching distance (across multiple trees, then across 100 resamplings) between a partition and the entire matrix was smaller (more similar). “Craniodental or postcranial $P$ value” reports the result of a Wilcoxon test on the 100 paired mean (across multiple trees) nearest neighbor matching distances between craniodental and postcranial subsamples and the entire matrix. Theropoda refs 1–29; Sauropodomorpha refs 30–46; Cerapoda refs 47–52; Ornithopoda refs 53–67; Thyreophora refs 68–81. *Sources*: Brown et al. (2011), Burns et al. (2011), Butler et al. (2011), Carballido et al. (2011), Eddy and Clarke (2011), Farke et al. (2011), Longrich (2011), Longrich et al. (2011), Pol et al. (2011a, 2011b), Santucci and De Arruda-Campos (2011), Allain et al. (2012), Fanti et al. (2012), Han et al. (2012), Ignacio Ruiz-Omenaca et al. (2012), McDonald (2012), Osi et al. (2012), Rubilar-Rogers et al. (2012), Senter et al. (2012a,b), Thompson et al. (2012), Araújo et al. (2013), Arbour and Currie (2013), Brusatte and Benson (2013), Cau et al. (2013), Coria et al. (2013), D’Emic (2013), Evans et al. (2013), Farke and Sertich (2013), Godefroit et al. (2013), Loewen et al. (2013), Mannion et al. (2013), McGarrity et al. (2013), Novas et al. (2013), Prieto-Marquez et al. (2013), Prieto-Marquez and Wagner (2013), Tschopp and Mateus (2013), Zanno and Makovicky (2013), Arbour et al. (2014), Barrett et al. (2014), Brusatte et al. (2014), Burns and Currie (2014), Carballido and Sander (2014), Choiniere et al. (2014), Farke et al. (2014), Foth et al. (2014), Gorscak et al. (2014), Lacovara et al. (2014), Lamanna et al. (2014), Lee et al. (2014), Li et al. (2014a, 2014b), Longrich (2014), Lu et al. (2014), Porfiri et al. (2014), Saegusa and Ikeda (2014), Sanchez-Hernandez and Benton (2014), Tortosa et al. (2014), Xing et al. (2014), Zhou et al. (2014), Boyd (2015), Boyd and Pagnac (2015), Carballido et al. (2015), Evans and Ryan (2015), Evers et al. (2015), Fanti et al. (2015), He et al. (2015), Hu et al. (2015), Ignacio Canale et al. (2015), McPhee et al. (2015), Norman (2015), Parsons and Parsons (2015), Rauhut et al. (2015), Shibata et al. (2015), Wang et al. (2015a, 2015b), Wilson and Allain (2015), Xing et al. (2015). All data matrices were analyzed by their original authors using maximum parsimony. Those marked with an asterisk in the table were additionally analyzed using Bayesian inference.

### Measuring Homoplasy

The ensemble Consistency Index (CI) (Kluge and Farris 1969) is a commonly used and well-characterized index of homoplasy and was calculated here to compare levels of homoplasy across partitions. However, the CI suffers from well-documented drawbacks, notably its correlation with the number of characters and taxa in the data set (Archie 1989; Mounce et al. 2016). We remove these biases empirically here, using the residuals from regression analyses of CI on both matrix dimensions. In addition, we report the ensemble retention index (RI) (Kluge and Farris 1969) as a measure of retained synapomorphy. All indices were calculated in PAUP* 4.0a.154 for Macintosh (Swofford 2003).

### Statistical Tests for Congruence

**Figure 2.**
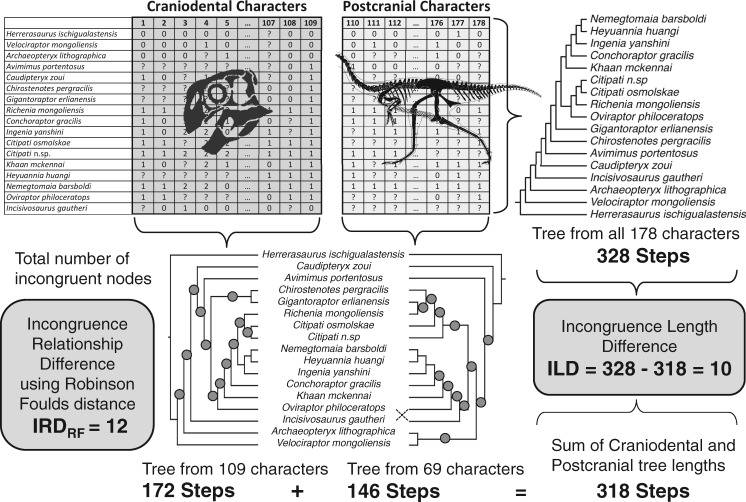
Calculation of the ILD and the IRD for the cranial and postcranial character partitions in the data of Fanti et al. (2012). Parsimony analysis of all 178 characters together yields a single MPT of 328 steps. Analysis of 109 craniodental characters alone yields an MPT length of 172 steps, while 69 postcranial characters alone yield an MPT length of 146 steps. The sum of these partitioned lengths ($172 + 146 =318$) is less than the length of the global MPT by 10 steps ($328 - 318 = 10$). This discrepancy is the incongruence length difference (ILD$=10$). The partitioned trees imply different relationships, and the magnitude of this difference can be measured using a diversity of tree-to-tree distance metrics. The RF or symmetrical difference distance (Robinson and Foulds 1981) is among the most widely applied, and is calculated as the sum of the number of internal tree nodes that are present in one tree but not the other. The IRD$_{RF}$ is therefore the incongruence relationship difference measured using the RF distance. The RF distance has the disadvantage that it can saturate quickly. Numerous other tree-to-tree distance metrics are available, and we also implement the matching distance here (MD) (Lin et al. 2012), to yield the IRD$_{matching}$. The ILD, IRD$_{RF}$, and IRD$_{matching}$ each enables a test of incongruence, implemented by randomly partitioning the data set into character sets of the same size as the original (here, 109 and 69) and recalculating the metric. This is repeated a large number of times to yield a null distribution for randomized metric values, and the value for the original partition is compared with this in order to yield an empirical $P$ value. Because the ILD, IRD$_{RF}$, and IRD$_{MD}$ all measure different things (tree length vs. different aspects of tree shape and relationships), the results of these tests do not invariably coincide. Silhouette modified from: https://en.m.wikipedia.org/wiki/File:Gigantoraptor_skeletal.png.

The ILD test (Mickevich and Farris 1981; Farris et al. 1995) is a widely implemented partition homogeneity test based upon the difference in MPT length for a matrix when analyzed as a whole, and the sum of MPT lengths for the partitions of the matrix analyzed in isolation (MPTs). More formally, the ILD for a bipartitioned matrix is given by $L_{AB} - (L_{A }+L_{B})$, where $L_{AB}$ is the optimal tree length (in steps) from the analysis of the entire matrix (the total evidence analysis), and $L_{A}$ and $L_{B}$ are the optimal tree lengths for partitions A and B analyzed independently (Fig. 2). This ILD is compared with a distribution of ILD values (here, 999) for random bipartitions of the matrix in the same proportions as the original, and a $P$ value is derived from the fraction of these as large or larger than the original. The ILD test has been criticized on philosophical grounds, and because it has a high Type I error rate (Dolphin et al. 2000; Barker and Lutzoni 2002; Ramirez 2006; Sansom et al. 2017). However, it remains very widely applied (Mounce et al. 2016) and is used here as a measure of matrix partition incongruence rather than as a criterion for combining those partitions.

In addition to the ILD test, we also implemented the incongruence relationship difference (IRD) test of Ruta and Wills (2016) and Mounce et al. (2016). This is analogous to the ILD test in that a measure of incongruence for the original data partition is compared with a distribution of incongruence values for a large number of random partitions. However, whereas for the ILD incongruence is measured in terms of additional tree length, a tree-to-tree distance index is used for the IRD. Many such indices are available, but here we used the symmetrical-difference (Robinson–Foulds [RF]) distance (IRD$_{RF})$ (Robinson and Foulds 1981) and matching (MD) distance (IRD$_{matching})$ (Lin et al. 2012). The RF distance is well characterized and widely applied but prone to saturation. In particular, transplanting a single leaf can cause the RF distance to maximize in a tree of any size. Indices of distance based upon tree editing, such as the maximum agreement subtree distance (Goddard et al. 1994; de Vienne et al. 2007) are computationally intensive. The matching distance has the advantages that it is formally metric, not prone to saturation, behaves intuitively and can be computed in polynomial time (Lin et al. 2012). It is unusual for a single MPT to result from a parsimony search, and we therefore followed Mounce et al. (2016) in calculating the mean nearest neighbor distance (NND) between each tree resulting from one partition and the most similar tree in the other partition. In addition, we calculated the distances between 50% majority rule (plus compatible groupings) trees for the two partitions, although we caution that these offer poor summaries of the differences between sets of trees (Mounce et al. 2016). IRD tests were initially based upon 99 random partitions of the data (c.f. 999 for the computationally much faster ILD).

All parsimony searches were implemented using 25 random additions of taxa, followed by tree bisection and reconnection branch swapping, and retaining 10 trees at each step. We also condensed the resulting MPTs by collapsing branches with a minimum length of zero (equivalent to Goloboff’s “amb-”; Goloboff et al. 2008) and removing all but one of any consequently identical trees. To expedite the searches, we limited the number of trees stored in memory to 100,000, and for the IRD tests we calculated nearest neighbor tree-to-tree distances based upon no more than 1000 MPTs from each partition. Consensus trees were calculated from all MPTs, up to the 100,000 limit. All analyses were carried out in PAUP* 4.0a.154 for Macintosh (Swofford 2003), and with the use of scripts (see Supplementary Material S3 available on Dryad) that produced batch files for PAUP* and summarized the logged output.

### Determining Whether Craniodental or Postcraniodental Characters Yield Trees More Similar to Those from the Entire Data Matrix

In cases where the tree(s) inferred from craniodental and postcranial characters differ (and especially where these differences are significant), it is reasonable to ask which tree is likely to be most accurate. Unfortunately, there are no objective tests of phylogenetic accuracy, except in those exceptional cases where phylogeny is known (e.g., laboratory cultures or simulated data sets). One approach for extant taxa (Sansom et al. 2017) is to determine the congruence of suites of morphological characters with a robustly supported molecular tree for the same taxa (an independent data source). However, this assumes that the molecular tree is likely to offer the best approximation of the truth: a standpoint defended in many quarters (Scotland et al. 2003; Olmstead and Scotland 2005; Wortley and Scotland 2006; Zou and Zhang 2016). Here, we ask whether trees from the craniodental or postcranial data partition are most similar to those derived from the entire morphological data matrix, with the underlying assumption that the total evidence tree is likely to be the most accurate (Kluge 1989; Gatesy et al. 1999; Gatesy and Arctander 2000). A straightforward approach would be to calculate mean nearest neighbor tree-to-tree distance metrics for the craniodental to entire tree sets versus the postcranial to entire tree sets. However, *all other things being equal*, a larger partition contributes more characters to the entire matrix than a smaller one. In cases where the optimal trees for the two partitions differed, the larger partition might therefore be expected to yield trees more similar to those from the entire data set. The difference in character numbers in the partitions could be overcome by differential weighting of characters, but the tree-to-tree distance metrics utilized here are sensitive to the reductions in resolution that are likely as the character: taxon ratio declines (and this is not overcome by weighting). We therefore adopted a resampling approach, repeatedly jack-knifing characters at the sample size of the smallest partition $(n)$ from both the larger partition and the entire matrix. For the entire matrix, we randomly jack-knifed the same number of characters ($n/2$) from both the craniodental and postcranial partition, such that neither was favored with a larger sample size. Where $n$ was an uneven number, we alternately sampled the “additional” character from either partition. For each of 100 resamplings, we then calculated the mean NND between craniodental and entire trees, and the mean NND between postcranial and entire trees. We include scripts for this procedure as Supplementary Material S4 available on Dryad. We report the median of these 100 comparisons (which partition is closest to the entire), as well as Mann–Whitney U test results to approximate a $P$ value at which to reject the null hypothesis that the medians of these distances are the same.

### Stratigraphic Congruence of Trees from Craniodental and Postcranial Partitions

Stratigraphic congruence may be used as an ancillary criterion for choosing (i.e., filtering) between otherwise equally optimal trees (Wills 1998; Wills et al. 2009; O’Connor and Zhou 2013; O’Connor and Wills 2016), or alternatively it can be used alongside morphological and molecular character data to find the optimal trees overall (i.e., to find trees that may be suboptimal for morphology and/or molecules considered alone) (Wagner 2000; Fisher 2008; Arregoitia et al. 2013; Lee and Yates 2018). However, this is only defensible where the fossil record is relatively complete, or at least where the order of first occurrences for lineages is preserved with reasonable fidelity. Previous analyses of cladograms of non-avian dinosaurs (Wills et al. 2008, 2009) reveal particularly strong stratigraphic congruence, suggesting that this may be a suitable ancillary test of accuracy. We therefore calculated the GER (Wills 1999), MSM* (Siddall 1998; Pol and Norell 2001), and SCI (Huelsenbeck 1994) for all of the MPTs from each partition (craniodental or postcranial) of each data set. However, all three indices are biased by tree balance, amongst other factors (Hitchin and Benton 1997). In addition, therefore, we calculated the GER* (Wills et al. 2008) based upon 10,000 random reassignments of stratigraphic range data to each tree. This is less sensitive to a number of potentially biasing factors (O’Connor et al. 2011; O’Connor and Wills 2016) and is therefore our preferred index. Scripts are available as Supplementary Material S5 available on Dryad.

## Results

### Craniodental and Postcranial Characters Contain Similar Levels of Homoplasy and Retain Similar Amounts of Synapomorphy

Statistics and test results for all 81 data sets are given in Table 1, and we distil these further in Table 2. We found no significant difference in the level of craniodental/postcranial ensemble CI across all 81 data sets (Wilcoxon test paired $V=$ 1637.5, $P=$ 0.9350). With similar medians (100 and 97) and overall distributions, the number of craniodental and postcranial characters were not significantly different ($V=$ 1342, $P$ value = 0.1343). We therefore compared the residual CI values from a linear regression of CI on the log of the number of characters and the log of the number of taxa, plus their interaction. This model was significant overall ($P <$ 2.2e-16), but none of the individual slope terms was significant ($P >$ 0.18 in all cases). Residuals from this model likewise showed no significant difference between partitions (Wilcoxon $V=$ 1595, $P=$ 0.760). Likewise, we found no significant difference in the level of craniodental/postcranial ensemble RI across all 81 data sets ($V=$ 1826, $P=$ 0.437). Similarly, the residuals from the regression of RI onto the number of taxa, number of characters and their interaction ($P=$ 2.168e-07, but with no significant slopes for individual terms; $P >$ 0.460) also showed no difference between partitions (Wilcoxon $V=$ 1815, $P=$ 0.468). Neither partition of the data can be deemed superior on the basis of these ensemble indices of internal consistency and retained synapomorphy.

**Table 2.** Summary of matrix partitions and results of tests

	All matrices	Theropoda	Sauropodomorpha	Cerapoda	Ornithopoda	Thyreophora	G	$P$ value
No. matrices	81	29	17	6	15	14	—	—
Median number craniodental/postcranial characters	100/97	122/177	95/160	96.5/37.5	100/53	125.5/93.5	—	—
Median craniodental/postcranial CI	0.597/0.587	0.629/0.585	0.669/0.582	0.742/0.808	0.591/0.599	0.504/0.548	—	—
Median craniodental/postcranial RI	0.771/0.731	0.768/0.710	0.778/0.668	0.819/0.848	0.777/0.767	0.688/0.732	—	—
Craniodental/postcranial most similar to entire (NND+matching)$^{a}$	39/42	13/16	4/13	5/1	10/5	7/7	—	—
f $P <$ 0.05 ILD	27	14	7	0	2	4	11.3810	**0**.**0226**
f $P <$ 0.05 IRD$_{{\rm NND}+{\rm RF}}$	45	21	9	3	5	7	6.7962	0.1471
f $P <$ 0.05 IRD$_{{\rm MR}+{\rm RF}}$	39	17	9	1	5	7	5.4241	0.2465
f $P <$ 0.05 IRD$_{{\rm NND}+{\rm matching}}$	41	20	12	4	3	2	21.6231	**0.0002**
f $P <$ 0.05 IRD$_{{\rm MR}+{\rm matching}}$	33	18	9	2	3	1	17.6366	**0.0015**

$^{a}$Craniodental/Postcranial most similar to entire (NND+matching). This indicates the number of matrices for which the craniodental/postcranial partitions yielded trees most similar to the entire matrix. Both partitions and the entire matrix were repeatedly (x100) resampled at the sample size (number of characters) of the smaller partition, and most parsimonious trees were inferred from all three samples of characters. For each resampling, the mean matching distance between nearest neighbors was used to determine which partitioned tree(s) (craniodental or postcranial) were most similar to the tree(s) from the subsampled entire matrix. The mean of these distances across all random resamplings was then used to determine which partition (craniodental or postcranial) yielded trees most similar to that from the entire matrix overall. The last five rows of the table indicate the frequency with which partitions yield trees that are significantly different (in bold, with $P <$ 0.05) for the ILD and variants of the IRD test. IRD tests are based either upon the mean tree-to-tree distances between nearest neighbors (NND) or the distance between majority rule (plus compatible grouping) trees (MR). The tree-to-tree distance metric used is either the Robinson–Foulds (symmetrical difference) distance (RF) or the Matching distance (Matching).

### Half of Craniodental and Postcranial Data Partitions Yield Significantly Different Trees

**Figure 3.**
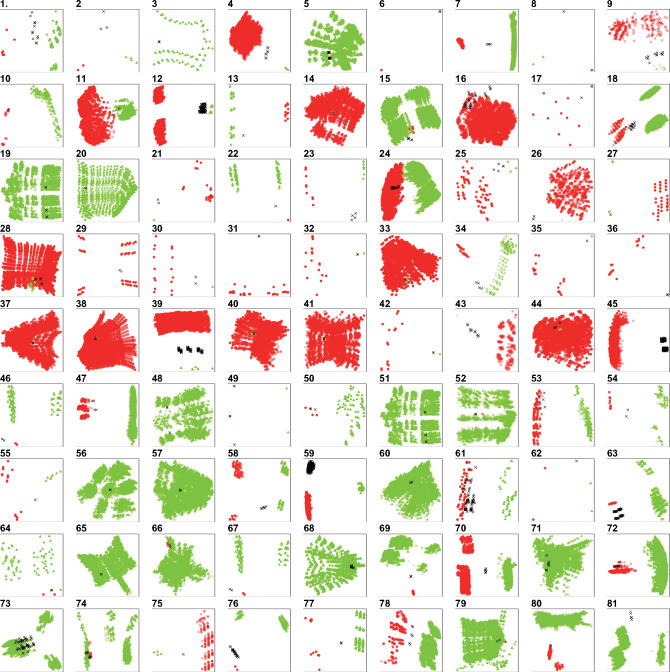
Indicative, two-dimensional nonmetric multidimensional scaling (NMDS) of tree spaces derived from RF distances for each of our 81 data matrices. Circles indicate MPTs inferred from craniodental characters, triangles indicate MPTs inferred from postcranial characters and crosses denote MPTs from both partitions analyzed simultaneously. RF distance matrices were calculated using *RF.dist* in the *Phangorn* package in R, and NMDS plots were generated from these matrices using *isoMDS* in *MASS*. Source papers are as follows: *Theropoda* 1. Allain et al. (2012), 2. Araújo et al. (2013), 3. Brusatte and Benson (2013), 4. Brusatte et al. (2014), 5. Canale et al. (2015), 6. Cau et al. (2012), 7. Choiniere et al. (2014), 8. Eddy and Clarke (2011), 9. Evers et al. (2015), 10. Fanti et al. (2012), 11. Farke and Sertich (2013), 12. Foth et al. (2014), 13. Godefroit et al. (2013), 14. Hu et al. (2015), 15. Lamanna et al. (2014), 16. Lee et al. (2014), 17. Li et al. (2014a), 18. Loewen et al. (2013), 19. Longrich et al. (2011), 20. Lu et al. (2014), 21. Novas et al. (2013), 22. Parsons and Parsons (2015), 23. Porfiri et al. (2014), 24. Sanchez-Hernandez and Benton (2014), 25. Senter et al. (2012), 26. Tortosa et al. (2014), 27. Wang et al. (2015), 28. Zanno and Makovicky (2013), 29. Zhou et al. (2014), *Sauropodomorpha* 30. Carballido and Sander (2014), 31. Carballido et al. (2015), 32. D’Emic (2013), 33. Fanti et al. (2015), 34. Gorscak et al. (2014), 35. Lacovara et al. (2014), 36. Li et al. (2014b), 37. Mannion et al. (2013), 38. McPhee et al. (2015), 39. Pol et al. (2011a), 40. Rauhut et al. (2015), 41. Rubilar-Rogers et al. (2012), 42. Saegusa and Ikeda (2014), 43. Santucci and De Arruda-Campos (2011), 44. Tschopp and Mateus (2013), 45. Wilson and Allain (2015), 46. Xing et al. (2015), *Cerapoda* 47. Evans and Ryan (2015), 48. Farke et al. (2011), 49. Farke et al. (2014), 50. Han et al. (2015), 51. Longrich (2011), 52. Longrich (2014), *Ornithopoda* 53. Boyd and Pagnac (2015), 54. Boyd (2015), 55. Brown et al. (2011), 56. Evans et al. (2013), 57. Godefroit et al. (2012), 58. He et al. (2015), 59. McDonald (2012), 60. McGarrity et al. (2013), 61. Norman (2015), 62. Norman et al. (2011), 63. Prieto-Marquez and Wagner (2013), 64. Prieto-Marquez (2014), 65. Prieto-Marquez et al. (2013), 66. Shibata et al. (2015), 67. Xing et al. (2014), *Thyreophora* 68. Arbour and Currie (2013), 69. Arbour et al. (2014), 70. Barrett et al. (2014), 71. Burns and Currie (2014a), 72. Burns and Currie (2014b), 73. Burns et al. (2011), 74. Butler et al. (2011), 75. Coria et al. (2013), 76. Godefroit et al. (2014), 77. Han et al. (2012), 78. Osi et al. (2012), 79. Pol et al. (2011b), 80. Ruiz-Omenaca et al. (2012), 81. Thompson et al. (2012).

For visualization purposes, the trees inferred from the craniodental and postcranial partitions of each data set have been plotted in a two-dimensional, nonmetric multidimensionally scaled tree space derived from RF distances, using the *RF.dist* function in *Phangorn* (Schliep 2011) and the *iso.MDS* function in the *MASS* package (Venables and Ripley 2002) for $R$ (Fig. 3). We note that such spaces, being nonmetric, are unsuitable as the basis for metric tests of partition homogeneity, but they do permit the differences between sets of trees to be figured impressionistically. Previous work on a broad sample of tetrapod matrices revealed significant incongruence between craniodental and postcranial character partitions about one time in three, as measured by both the incongruence relationship difference (IRD) test of Ruta and Wills (2016) and the ILD test (Mickevich and Farris 1981; Farris et al. 1995). Here, we report that 50% of dinosaur matrices yielded significantly ($P <$ 0.05) incongruent trees according to the IRD test for nearest neighbors using matching distances (IRD$_{NND+matching})$ and 54% for the IRD test using the RF distances (Robinson and Foulds 1981) (IRD$_{NND+RF})$. Moreover, the IRD$_{NND+matching}$ and IRD$_{NND+RF}$ values were significantly correlated ($r_{s}=$ 0.649, $P=$ 8.999e-14). We therefore also report the results of IRD tests using majority rule consensus trees derived from up to 10,000 optimal source trees. Inevitably, consensus trees cannot reflect accurately the diversity of relationships within a set of source trees (Mounce et al. 2016) but they do permit tests that incorporate all source trees more readily. The consensus results were closely similar to those for the nearest neighbor tests: 63% of matrices were significantly incongruent using IRD$_{MR+matching}$ and 60% were incongruent using IRD$_{MR+RF}$. Moreover, the consensus results were strongly and significantly correlated with the NND results for both the IRD$_{MR+matching }(r_{s}=$ 0.863, $P=$ 2.2e-16) and IRD$_{MR+RF }(r_{s}=$ 0.837, $P=$ 2.2e-16). The rate of significance (33% at $P <$ 0.05) for the ILD test was lower than that for variants of the IRD, and similar to that observed for tetrapods overall (Mounce et al. 2016).

Mounce et al. (2016) tested empirically whether the rate of null rejection was influenced by several data matrix parameters. Here, we used logistic regression to determine the outcome of each of our five partition homogeneity tests (significant or not with $P <$ 0.05) as a function of the overall number of taxa, overall number of characters (both partitions), the difference in partition size (scaled relative to the total number of characters in both partitions), the absolute size of the smaller partition, the percentage of missing data in the entire matrix, and the difference in the percentage of missing data between partitions. Results are summarized in the Supplementary Material S6 available on Dryad. For the ILD, both the number of taxa and the total number of characters were retained in the minimum adequate model (MAM) selected by sequentially deleting the least significant independent variables. For the IRD$_{NND+RF }$(nearest neighbor RF distances between groups of trees), only the size of the smallest partition was retained in the MAM. For the IRD$_{MR+RF}$ (RF distances between majority rule trees), the total number of taxa and the overall percentage of missing data were retained in the MAM. However, we strongly caution against the use of consensus trees in the IRD test. Hence, as reported by Mounce et al. (2016), tests based upon symmetrical differences (Robinson and Foulds 1981) are influenced, at least in some measure, by partition dimensions. For the IRD$_{NND+matching}$ (based upon nearest neighbor matching distances, and our preferred metric), the number of taxa, total number of characters, and the overall percentage of missing data were retained in the MAM, with all being significant ($P <$ 0.031). For the IRD$_{MR+matching}$ (based upon matching distances between majority rule trees), only the total percentage of missing data was retained.

### Levels of Incongruence Vary Significantly across Major Dinosaur Groups

**Figure 4.**
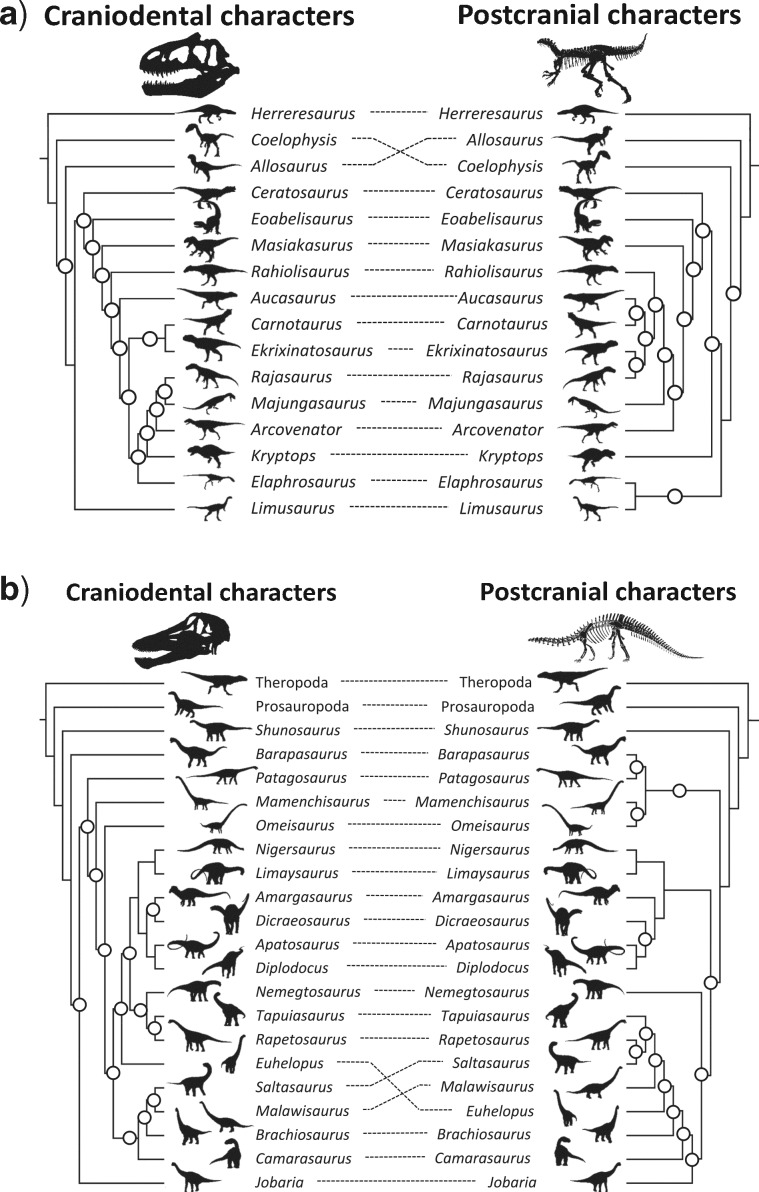
Example tanglegrams for two groups of Saurischia. All trees are majority rule trees, plus compatible groupings. We do not necessarily recommend the use of majority rule trees in calculating IRD statistics (although we summarize these IRD$_{MR}$ calculations in Tables 1 and 2), because they can be far from the centroid of tree space (Mounce et al. 2016). Rather, we advocate the use of mean distances between nearest neighbors in the two sets of trees for comparison. The left-hand tree in each panel is derived from craniodental characters, while the right-hand tree is derived from postcranial characters. Circled internal nodes are those present in one tree but not the other, and are tallied to give the RF or symmetrical difference distance (Robinson and Foulds 1981). **a**) Tanglegram for Theropoda using data from Tortosa et al. (2014). (ILD $P=$ 0.017; IRD$_{NND+RF} P=$ 0.01; IRD$_{NND+matching} P=$ 0.01; IRD$_{MR+RF} P=$ 0.01; IRD$_{MR+matching} P $= 0.01). **b**) Tanglegram for Sauropoda using data from Gorscak et al. (2014). (ILD $P=$ 0.074; IRD$_{NND+RF} P=$ 0.02; IRD$_{NND+matching} P=$ 0.02; IRD$_{MR+RF} P=$ 0.02; IRD$_{MR+matching} P=$ 0.01). All silhouettes are original drawings by Yimeng Li.

**Figure 5.**
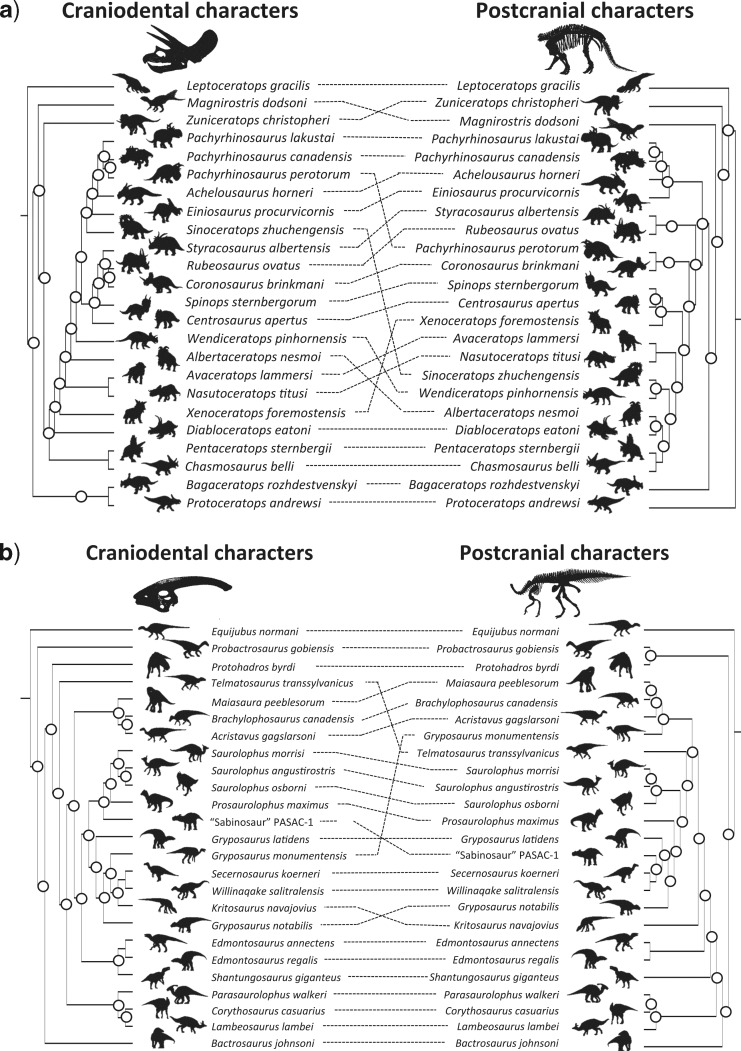
Example tanglegrams for two groups of Ornithischia. All trees are majority rule trees, plus compatible groupings. We do not necessarily recommend the use of majority rule trees in calculating IRD statistics (although we summarize these IRD$_{MR}$ calculations in Tables 1 and 2), because they can be far from the centroid of tree space (Mounce et al. 2016). Rather, we advocate the use of mean distances between nearest neighbors in the two sets of trees for comparison. The left-hand tree in each panel is derived from craniodental characters, while the right-hand tree is derived from postcranial characters. Circled internal nodes are those present in one tree but not the other, and are tallied to give the RF or symmetrical difference distance (Robinson and Foulds (1981)). **a**) Tanglegram for Ceratopsia using data from Evans and Ryan (2015). (ILD $P=$ 0.078; IRD$_{NND+RF} P=$ 0.04; IRD$_{NND+matching} P=$ 0.01; IRD$_{MR+RF}$$P=$ 0.03; IRD$_{MR+matching} P=$ 0.01). **b**) Tanglegram for Hadrosaurida using data from Prieto-Marquez (2014). (ILD $P=$ 0.088; IRD$_{NND+RF} P=$ 0.34; IRD$_{NND+matching} P=$ 0.12; IRD$_{MR+RF} P=$ 0.83; IRD$_{MR+matching} P=$ 0.37). All silhouettes are original drawings by Yimeng Li.

**Figure 6.**
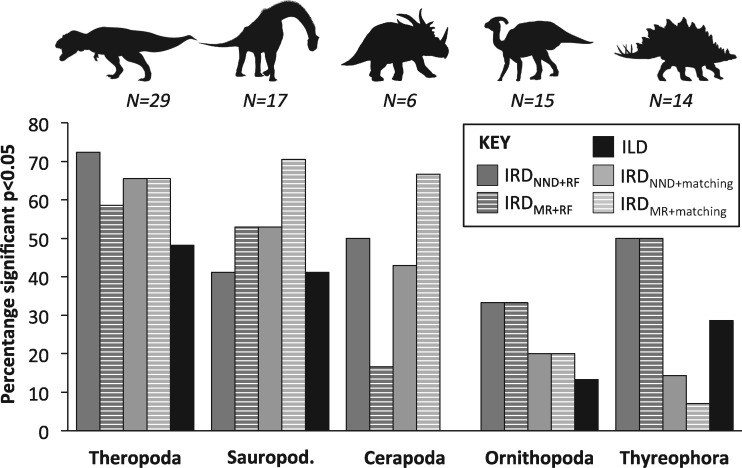
Summary of results of ILD and variants of the IRD tests, partitioned by major taxonomic group. Bars denote the percentage of data sets for which $P <$ 0.05. “RF” in the subscript denotes IRD tests utilizing the symmetrical-difference distance of Robinson and Foulds (1981), while “matching” in the subscript denotes tests utilizing the Matching distance of Linn et al. (2012). Comparisons are either made using majority rule consensus trees (MR), or the mean NND between each tree in one set and its nearest neighbor in the other. Silhouettes: https://publicdomainvectors.org/en/free-clipart/Tyrannosaurus-Rex-silhouette/63931.html; https://pixabay.com/vectors/silhouette-dinosaur-brachiosaurus-3464840/; https://hu.wikipedia.org/wiki/Fájl:Human-styracosaurus_size_comparison.png; https://publicdomainvectors.org/en/free-clipart/Dino-silhouette-image/63930.html; https://publicdomainvectors.org/en/free-clipart/Stegosaurus-shadow/63906.html.

Of our 46 saurischian matrices, 31 (67%) showed significant ($P <$ 0.05) incongruence using IRD$_{NND+matching}$, compared with 9 from 35 (26%) ornithischian matrices: a significant difference in the rate of null rejection (likelihood ratio test: $G=$ 20.1841, $P=$ 0.00046). A similarly significant difference was observed for the IRD$_{MR+matching}$ ($G=$ 19.3857, $P=$ 0.00066). For the IRD using RF distances, by contrast, differences across saurischian and ornithischian matrices were nonsignificant (IRD$_{NND+RF}$ G = 6.7962, $P=$ 0.14706: IRD$_{MR+RF} \quad G=$ 5.4241, $P=$ 0.24648). At a finer taxonomic level (specifically assigning trees to Theropda [e.g., Fig. 4a], Sauropodomorpha [e.g., Fig. 4b], Cerapoda [e.g., Fig. 5a], Ornithopoda [e.g., Fig. 5b], and Thyreophora [Table 2]), there were highly significant differences in the rate of null rejection using the matching distance ($P=$ 0.00046 for the IRD$_{NND+matching}$: $P=$ 0.00066 for IRD$_{MR+matching})$ but no differences using the symmetrical difference (RF) variants of the tests (Table 2) (Fig. 6). Rates of partition incongruence are relatively high in the Sauropodomorpha (53% for the IRD$_{NND+matching})$, Theropoda (66%), and Ceropoda (43%) compared with the Ornithopoda (20%) and Thyreophora (14%). A similar hierarchy of outcomes pertained for the other tests.

### Craniodental and Postcranial Characters Produce Trees Equally Similar to the Entire Data Set, but with Strong Biases across Major Groups

Our second set of tests sought to determine whether the MPTs from the entire matrix were more similar to those from the craniodental or postcranial partitions. The results from these were in strong agreement. Considering the NNDs for matching distances across all 81 matrices, 42 were closer (using the matching distances for nearest neighbors: NND+matching) to the postcranial partition, while 39 were closer to the craniodental partition (a nonsignificant bias: binomial test $P=$ 0.8243). Within Saurischia and Ornithischia, however, the biases were highly significant, but in opposite directions (G = 6.4242, $P=$ 0.0113). For Saurischia, trees from the entire data set were most often more similar to those from the postcranial partition (29 cases) than to those from the cranium (17 cases) (binomial $P=$ 0.1038). For Ornithischia, by contrast, trees from the whole data set were more often most similar to those from the craniodental partition (22 cases) than to those from the postcranium (13 cases) ($P=$ 0.1755). When partitioned into five groups as above, there was also a significant difference in which partition was most similar to the entire matrix across groups (G = 8.7347, $P=$ 0.0062), with the bias for Sauropodomorpha (13 postcranial vs. 4 craniodental) being the most striking. Similar findings were made for the other three tests (Tables 1 and 2).

These biases were much less marked if comparisons were restricted to only those data sets for which there was a significant difference ($P <$ 0.05) between the distributions of distances (entire to craniodental vs. entire to postcraniodental) according to the Mann–Whitney U test. Considering NND+matching distances, 31 data sets favored the craniodental partition and 33 the postcranial partition (sign test $P=$ 0.9007). Moreover, there was no longer a significant bias in favor of postcranial characters for the Saurischia (23 out of 33: $P=$ 0.0308) or in favor of craniodental characters for the Ornithischia (17 out of 31: $P=$ 0.7201) (overall G for Saurischia and Ornithischia = 3.9845, $P=$ 0.0459). For the partition into Theropda, Sauropodomorpha, Cerapoda, Ornithopoda, and Thyreophora, we marginally retained the null hypothesis that groups behave identically ($G=$ 9.2929, $P=$ 0.0542). Similar findings for the other three tests are summarized in Table 2.

### There is No Difference in the Stratigraphic Congruence of Trees Inferred from Craniodental or Postcranial Data, with the Exception of Sauropodomorpha

We present indices of stratigraphic congruence for cranial and postcranial partitions of all 81 data matrices (162 partitions) in Supplementary Materials S7 available on Dryad. Across all matrices, we observed no significant differences in stratigraphic congruence for trees inferred from cranial versus postcranial data, whether using the GER* (craniodental median $\tilde{x} =$ 0.879, postcranial $\tilde{x} =$ 0.942, $V=$ 2009, $P=$ 0.101), GER (craniodental $\tilde{x} =$ 0.781, postcranial $\tilde{x} =$ 0.804, Wilcoxon $V=$ 1739, $P=$ 0.570), MSM* (craniodental $\tilde{x} =$ 0.320, postcranial $\tilde{x} =$ 0.312, $V=$ 1665, $P=$ 0.831), or SCI (craniodental $\tilde{x} =$ 0.500, postcranial $\tilde{x} =$ 0.538, $V=$ 1980, $P=$ 0.133) (Table 3). We note that the GER, MSM*, and particularly the SCI are all influenced by a number of undesirable factors, including tree balance (Siddall 1996, 1997; Pol et al. 2004). The GER* is our preferred index of congruence, since it is relatively impervious to such biases (O’Connor and Wills 2016). Hence, while postcranial character partitions are more congruent than craniodental partitions overall according to all indices except the MSM*, none of these differences is significant.

**Table 3.** Summary of stratigraphic congruence indices, tree balance and percentage resolution for sets of most parsimonious trees (MPTs) across all Dinosauria and major subclades.

	GER*			GER			MSM*		
	Median		$P$ value	Median		$P$ value	Median		$P$ value
	Cran.	Post.		Cran.	Post.		Cran.	Post.	
All Dinosauria	0.879	0.942	0.1013	0.781	0.804	0.5698	0.320	0.312	0.8310
Theropoda	0.846	0.942	0.5337	0.767	0.772	0.2297	0.303	0.317	0.2297
Sauropodomorpha	0.727	0.906	**0.0000**	0.750	0.826	**0.0000**	0.330	0.437	**0.0000**
Cerapoda	0.729	0.747	1.0000	0.737	0.587	0.2807	0.268	0.206	0.5896
Ornithopoda	0.994	0.969	0.1671	0.883	0.855	0.0637	0.361	0.313	0.0413
Thyreophora	0.969	0.980	0.7798	0.814	0.775	0.0256	0.276	0.214	0.0103

	SCI			Colless’s Index			Percentage resolution		
	Median		$P$ value	Median		$P$ value	Median		$P$ value
	Cran.	Post.		Cran.	Post.		Cran.	Post.	
All Dinosauria	0.500	0.538	0.1331	0.440	0.445	0.8066	94.25	94.20	0.3456
Theropoda	0.472	0.538	0.3692	0.416	0.476	0.4812	94.24	94.44	0.2763
Sauropodomorpha	0.431	0.531	**0.0001**	0.437	0.467	0.3060	92.93	94.87	**0.0000**
Cerapoda	0.750	0.752	0.8438	0.515	0.506	0.1563	89.98	85.79	0.3125
Ornithopoda	0.591	0.549	0.4212	0.466	0.438	0.6788	95.13	93.32	**0.0017**
Thyreophora	0.524	0.512	0.5614	0.392	0.355	0.1353	96.00	94.27	**0.0134**

We report the medians (across multiple data matrices) of the means (across all most parsimonious trees for each data matrix). $P$ values are recorded for paired Wilcoxon tests. Values in bold type where $P < 0.05$.

GER* = modified Gap Excess Ratio; GER = Gap Excess Ratio; MSM* = modified Manhattan Stratigraphic Metric; SCI = Stratigraphic Consistency Index; Colless’s index summarizes tree balance; percentage resolution is given by internal nodes/(terminals - 1) $\times $ 100.

We also summarize comparisons between partitions for each of our five major dinosaur groups (Table 3). Postcranial partitions had higher median GER* than their craniodental counterparts in Theropoda, Sauropodomorpha, Cerapoda, and Thyreophora, while the reverse was true in Ornithopoda. However, no indices revealed significant differences between craniodental and postcranial trees for Theropoda, Cerapoda, Ornithopoda, and Thyreophora, but all found a significant difference (paired Wilcoxon tests: $P \le$ 0.00004) for Sauropodomorpha. While the GER* is relatively insensitive to differences in tree balance, we note that there were no significant differences between median Colless’s index for craniodental versus postcranial trees, either across all dinosaurs or in any of the five subclades ($P >$ 0.135). The mean percentage resolution for cranial and postcranial trees was virtually identical across all 81 data sets ($\tilde{x}\,=$ 94.25 and 94.20, respectively: $P=$ 0.346). However, although absolute differences for our five constituent subclades were small (a maximum difference between medians of just 4.19% for Sauroppodomorpha), these differences were significant for Suropodomorpha (better resolved from postcranial characters; $P <$ 0.0001), Ornithopoda (better resolved from craniodental characters; $P=$ 0.0017), and Thyreophora (better from craniodental characters; $P=$ 0.0134).

We also tested for association between stratigraphic congruence (whether trees inferred from craniodental or postcranial characters were most congruent with stratigraphy, using each of GER*, GER, MSM*, and SCI) and consilience with total evidence (whether trees from craniodental or postcranial characters were most similar to trees from the entire data matrix using NND+matching distances, and correcting for sample size differences). We then tested each of the 2 $\times $ 2 contingency tables for association using likelihood ratio (G) tests, and results are summarized in Supplementary Materials S8 available on Dryad. Across all Dinosauria, we rejected the null hypothesis of no association, irrespective of the stratigraphic congruence index used ($P \le $ 0.00045). In other words, the data partition yielding trees most similar to the total evidence trees also tended to yield the most stratigraphically congruent trees. The same was also true for Theropoda considered in isolation ($P \le $ 0.00436 for all indices), and for Thyreophora according to the GER ($P=$ 0.02553), MSM* ($P=$ 0.02553), and SCI ($P=$ 0.00168) but not our preferred index, the GER* ($P=$ 0.08605). In contrast, Sauropodomorpha, Cerapoda, and Ornithopoda showed no association when considered in isolation ($P \ge $ 0.07792 in all cases).

## Discussion

### Implications for Dinosaur Phylogeny

Our analyses of 81 published matrices demonstrate empirically that the relationships of dinosaurs inferred from craniodental or postcranial characters in isolation differ significantly ($P <$ 0.05) from each other about half of the time. This is much more often than similar partitions for tetrapods in general (about 1 in 3: Mounce et al. 2016). At the same time, we find similar levels of homoplasy (as measured by the ensemble CI and retained synapomophy (RI) in craniodental and postcranial character partitions across all dinosaurs. Similarly, when character sample sizes are controlled, the relationships inferred from either partition are equally congruent with those from the entire matrix. Hence, there is no reason to prefer characters sampled from one partition versus another across dinosaurs as a whole, and we concur with general recommendations to sample characters widely from all anatomical regions in accordance with the principle of total evidence (Kluge 1989; Gatesy et al. 1999; Gatesy and Arctander 2000; Mounce et al. 2016). However, we also observe marked differences in levels of incongruence across major dinosaur groups, being significantly higher in Saurischia than Ornithischia.

Homoplasy is always a problem for phylogenetic inference, but is least troublesome when homoplastic states approximate to a random distribution across taxa (in which case it largely contributes noise). Homoplasy is more problematic when it is correlated across complexes of characters, especially when this occurs at higher frequencies. The morphological phylogeny of mammals appears to have been subject to such problems. Phylogenomic trees (Dolphin et al. 2000) overturned many of the groups (e.g., Ungulata and Insectivora) that had emerged from nearly all previous analyses of morphological characters over the preceding decades. Most problematic of all are cases where correlated homoplasy is concentrated within a particular region of the body, and especially where available data are limited to such regions. The teeth of mammals appear to be especially prone to such convergence (Goswami et al. 2011; Sansom et al. 2017), with many aspects of their form changing in concert and being controlled by a relatively small number of genes (Castelin et al. 2017). This is singularly unfortunate for those studying mammalian evolution, since the high preservation potential of teeth means that they dominate the fossil record. The fossil record of dinosaurs is also biased, with sauropodomorphs and theropods being known predominantly from their postcranial remains (skulls are often fragmentary or not recovered), and ceratopsians being more often described from their much more massive skulls (Evans and Ryan 2015). Our sample of matrices suggests that the number of coded characters strongly reflects these differences. However, it remains unclear whether this is solely a function of the available material, or whether systematists preferentially code or more finely atomize characters from these regions. Whatever the case, we do not find a higher concentration of homoplasy in either partition, whether across all dinosaurs or within major clades. Moreover, although craniodental and postcranial characters often yield significantly conflicting trees, we find no evidence that one partition is more likely to be congruent with the “total evidence” tree than the other.

### Interpreting Incongruence

The inference of significantly different trees from craniodental and postcranial character partitions can be understood in terms of divergent selective pressures operating on different regions of the body (Gould 1977; Kemp 2005; Lü et al. 2010). This results in different rates and patterns of character evolution (Mitteroecker and Bookstein 2007; Klingenberg 2008), in addition to distinct patterns of homoplasy. Anatomical modules are commonly recognized in studying the evolution of form (Mitteroecker and Bookstein 2007; Cardini and Elton 2008; Klingenberg 2008; Lü et al. 2010; Goswami et al. 2011; Hopkins and Lidgard 2012; Cardini and Polly 2013; Goswami et al. 2015), and it is reasonable to suppose that such modules will contain phylogenetic characters that are more congruent with one another than with characters from other modules (Clarke and Middleton 2008).

The tetrapod skull is variously decoupled from the skeleton of the body, both biomechanically and in terms of the selective pressures operating upon it (Ji et al. 1999; Mitteroecker and Bookstein 2007). However, this decoupling is particularly marked in the non-avian dinosaurs (Mounce et al. 2016). The long necks of sauropodomorphs effect the greatest biomechanical decoupling between the skull and the body, and sauropods unsurprisingly have one of the highest levels of significant incongruence. Ornithischia, by contrast, show much lower levels of incongruence overall.

Anatomical modules are typically envisaged as comprising physically proximate sets of characters or aspects of form. However, particular selective pressures might result in the coordinated evolution of suites of characters widely distributed across the body (Gardiner et al. 2011; Abourachid and Hoefling 2012; Godefroit et al. 2013). For example, a mode of predation or scavenging favored by many theropods entailed bracing a carcass with a back leg whilst ripping with powerful jaws and a strong neck (Rayfield 2004). This manner of feeding evolved in at least three large theropod clades, and entailed coordinated changes in the limbs and skull (Snively et al. 2006; Snively and Russell 2007a, 2007b; Hone and Rauhut 2010). Similarly, the massive skulls of many ceratopsians were braced into the body and pectoral girdle consistent with their ability to face and ward off predators, and this may have effected other coordinated changes in the vertebral column and back limbs.

In the infancy of molecular phylogenetics, trees were often inferred from single genes (Gatesy and Arctander 2000), and it was not uncommon for the trees derived from different genes to be strikingly at odds (Gatesy and Arctander 2000). In addition, molecular trees often differed markedly from those inferred from morphology. A more cautious, combinatorial approach initially evolved, therefore, along with partition tests designed to ensure homogeneity of signal (Mounce et al. 2016). This agglomerative approach may have been a function of the manner in which data originally became available, with systematists exploring incongruent signals at a fine level of granularity. The ascendance of phylogenomic analyses has brought its own bioinformatic challenges, but all approaches seek to derive trees from increasingly inclusive data sets. More philosophically, a consensus has emerged in favor of the principle of total evidence (Kluge 1989): the procedure by which all available character data are combined into a single matrix and analysis. One reason for this is the phenomenon of “hidden support” (Gatesy et al. 1999; Gatesy and Arctander 2000), whereby signals that are weak and therefore hidden within individual character partitions become dominant when all data are analyzed together. Various tests for partition homogeneity (Farris et al. 1994, 1995; Dolphin et al. 2000) will tend to return significant results in precisely those circumstances in which support is hidden, and such tests are therefore no longer commonly used to preclude the combination of data sources in this manner (Kluge 1989; Gatesy et al. 1999; Gatesy and Arctander 2000; Wahlberg et al. 2005; O’Leary and Gatesy 2008; Padial et al. 2010; Damgaard 2012; Mounce et al. 2016). However, while molecular systematics has retained and elaborated the notion that different suites of characters within large molecular matrices might be most effectively modeled with different rate parameters (e.g., different sets of genes or different codon positions), morphological data are rarely treated in this manner (but see Lanfear et al. 2017). Moreover, there is relatively little quantitative empirical data on the sorts of morphological characters that might be most useful for resolving relationships at different hierarchical levels within a phylogeny, or for radiations of different ages. There are principally two reasons for this. Firstly morphology is less likely to be constrained to evolve in a clock-like manner throughout a tree (or to change its rate in a manner amenable to modeling), although Drummond and Stadler (2016) have demonstrated cases where morphology is surprisingly clock-like. Secondly, despite the considerable utility of a number of searchable resources including Morphobank (O’Leary and Kaufman 2011), Treebase (Piel et al. 2009; Vos et al. 2012), and Phenoscape (**?**), morphological characters cannot be archived, retrieved, and coded in an automated and objective manner to produce iteratively larger matrices with the same ease that sequence data can. There have been strides in this direction utilizing machine reasoning (Dececchi et al. 2015; Dahdul et al. 2018), but in contrast to the situation for molecular sequence data, considerable taxon-specific expertise is still usually required to combine morphological character data. This is because systematists rarely atomize or code the same aspects of morphology in precisely the same manner, and sometimes express these using complex semantics. Despite considerable variation in rates of evolution and levels of homoplasy across morphological characters, trees are often inferred from relatively restricted character sets (Sanchez-Villagra and Williams 1998; Arratia 2009; Song and Bucheli 2010; Mounce et al. 2016). In fossil taxa, this may reflect preservational biases, particularly those favoring hard part preservation (Pattinson et al. 2015), and it is unfortunate that these biases appear to favor some of the most homoplastic characters (Sansom et al. 2010, 2017; Sansom and Wills 2013; Pattinson et al. 2015).

## Conclusions

1. Across our sample of 81 data sets, systematists have abstracted slightly fewer characters from the skull than the rest of the skeleton overall, although this difference is not significant ($V=$ 1342, $P=$ 0.1343). However, this masks particular biases in major dinosaur groups: markedly and significantly ($P <$ 0.02 in all cases) in favor of postcranial characters in Sauropodomorpha and Theropoda and in favor of craniodental characters in Ornithopoda, Thyreophora and Cerapoda.

2. The overall frequency of significant ($P <$ 0.05) incongruence between dinosaur trees inferred from craniodental and postcranial characters was about 50% for variants of the Incongruence Relationship Difference (IRD) test (Mounce et al. 2016; Ruta and Wills 2016). This was substantially higher than that previously reported (30%) for tetrapod clades in general. The ILD test reported significant incongruence in 33% of cases: comparable to the level seen across tetrapods hitherto (Mounce et al. 2016).

3. Despite the high frequency of incongruence overall, rates of significance were heterogeneously distributed across major dinosaur groups, being highest (a mean of 71% for variants of the IRD based upon NNDs) in Theropoda and lowest (27%) in Ornithopoda. We note that incongruence is greatest in those groups (Sauropodomorpha and Theropoda) in which the biomechanical decoupling between head and body is greatest. We also demonstrate that there are similar levels of homoplasy and retained synapomorphy between partitions overall. Incongruence therefore at least partly reflects differences in *patterns* of homoplasy between partitions, which may itself be a function of modularity and mosaic evolution.

4. A number of factors have been purported to influence the outcome of the ILD and IRD tests, notably the data matrix dimensions, relative partition sizes and the amount and distribution of missing entries (Mounce et al. 2016). We replicate these findings here, to which we add the absolute size of the smaller partition in the case of the IRD$_{NND+RF}$ test.

5. Tests to determine which partition (craniodental or postcranial) were most congruent with trees inferred from the entire character set were equivocal overall: equal numbers favored the two partitions once differences in sample size were controlled for. However, there were significant asymmetries in many groups, with the bias for Sauropodomorpha (13 postcranial vs. 4 craniodental) being the most striking. Across all 81 data matrices, the partition most congruent with the entire data set also tended to yield trees that were more stratigraphically congruent: a mutual consilience that is consistent with the hypothesis that those partitions yield more accurate trees. The same was unambiguously true (i.e., irrespective of the index of stratigraphic congruence used) for Theropoda considered in isolation.

6. Our results demonstrate clearly that phylogenies of dinosaurs inferred from craniodental and postcranial characters differ significantly much more often than expected. We therefore make the straightforward recommendation that characters should be sampled as broadly as possible from across all body regions. This accords with the theoretical principle of total evidence (Kluge 1989; Gatesy et al. 1999; Gatesy and Arctander 2000), as well as our empirical findings for tetrapods in general (Mounce et al. 2016) and mammals in particular (Sansom et al. 2017).

## Supplementary Material

Data available from the Dryad Digital Repository: http://dx.doi.org/10.5061/dryad.gxd2547gj.
